# Transformation of sludge Si to nano-Si/SiO_x_ structure by oxygen inward diffusion as precursor for high performance anodes in lithium ion batteries

**DOI:** 10.1186/s11671-018-2549-7

**Published:** 2018-05-03

**Authors:** Qiqi Hua, Dongyang Dai, Chengzhi Zhang, Fei Han, Tiezheng Lv, Xiaoshan Li, Shijie Wang, Rui Zhu, Haojie Liao, Shiguo Zhang

**Affiliations:** College of Materials Science and Engineering, Hunan University, Changsha, 410082 Hunan China

**Keywords:** Si sludge, Oxygen diffusion, Anode, Lithium-ion battery

## Abstract

Although several Si/C composite structures have been proposed for high-performance lithium-ion batteries (LIBs), they have still suffered from expensive and complex processes of nano-Si production. Herein, a simple, controllable oxygen inward diffusion was utilized to transform Si sludge obtained from the photovoltaic (PV) industry into the nano-Si/SiO_x_ structure as a result of the high diffusion efficiency of O inside Si and high surface area of the sludge. After further process, a yolk/shell Si/C structure was obtained as an anode material for LIBs. This composite demonstrated an excellent cycling stability, with a high reversible capacity (∼ 1250 mAh/g for 500 cycles), by void space originally left by the SiO_x_ accommodate inner Si expansion. We believe this is a rather simple way to convert the waste Si into a valuable nano-Si for LIB applications.

## Background

Lithium-ion batteries (LIBs) are the primary energy storage devices in our life [1]. Recently, the rapid development of electrical vehicles (EVs) has caused increasing demand for high-performance LIBs with a low price, high energy density, stability, and safety [2]. In this respect, various new active anode materials for LIBs are being developed; in particular, Si-related anode research has attracted considerable interest since it has the highest theoretical capacity of 4200 mAh/g. The main problem of Si is that the Li^+^ insertions/extractions produce a significant volume expansion (> 300%), which causes particle pulverization, lost electrical contact of the active materials, and a rapidly diminishing capacity [3]. Several well-designed Si structures or Si-based composites anodes for LIBs have been developed, such as Si nanowire [4], porous Si [5], Si/C/TiO_2_ double shell composite [6], granadilla-like Si/C composite [7], or binder free composite anode [8]. Despite many impressive achievements for Si anodes, most of the Si composite anodes were obtained using very expensive, low-yield commercial Si nanoparticles as starting material (http://www.sigmaaldrich.com/catalog/product/aldrich/795585?lang=zh&region=CN). Investigations of inexpensive, simply fabricated Si precursor for the anode of LIB are urgently needed.

The main applications of Si are in photovoltaic (PV) industry, as a wafer. For producing wafer, some Si from ingot is crushed into particles by grit and carried away in an aqueous slurry, eventually forming Si sludge. The total Si waste sludge is more than 100,000 MT yearly and increases nowadays. This Si sludge has dimensions with a D50 of approximately 1–2 μm [9]. Moreover, they have a larger active surface area than a bulk substrate for oxidation, which is favorable for SiO_x_ formation. The mass production of PV wafers causes considerable solid pollution of Si sludge; actually, this could be a good resource as an anode material for LIBs if an appropriate phase transformation could be performed.

Cui developed a novel method to obtain micrometer-level Si as rather stable anodes [10]; however, this process is still rather complicated, which involves Ni plating on Si particles and graphene CVD growth as indispensable steps. Solid Si sub-oxide, like SiO, has also been researched as a promising anode [11]. The reaction between SiO and Li^+^ in the first lithiation/delithiation produces a Li_2_O and Li_4_SiO_4_ matrix, which could diminish the huge volume variation of Si. Using metallurgical Si in ball milling with H_2_O can produce porosity-controlled SiO_x_, which has shown very promising electrochemical results [12]. Therefore, studying the role of O in fabricating a particular Si anode structure for an LIB is very important for future Si anode development.

## Methods

**Fig. 1:**
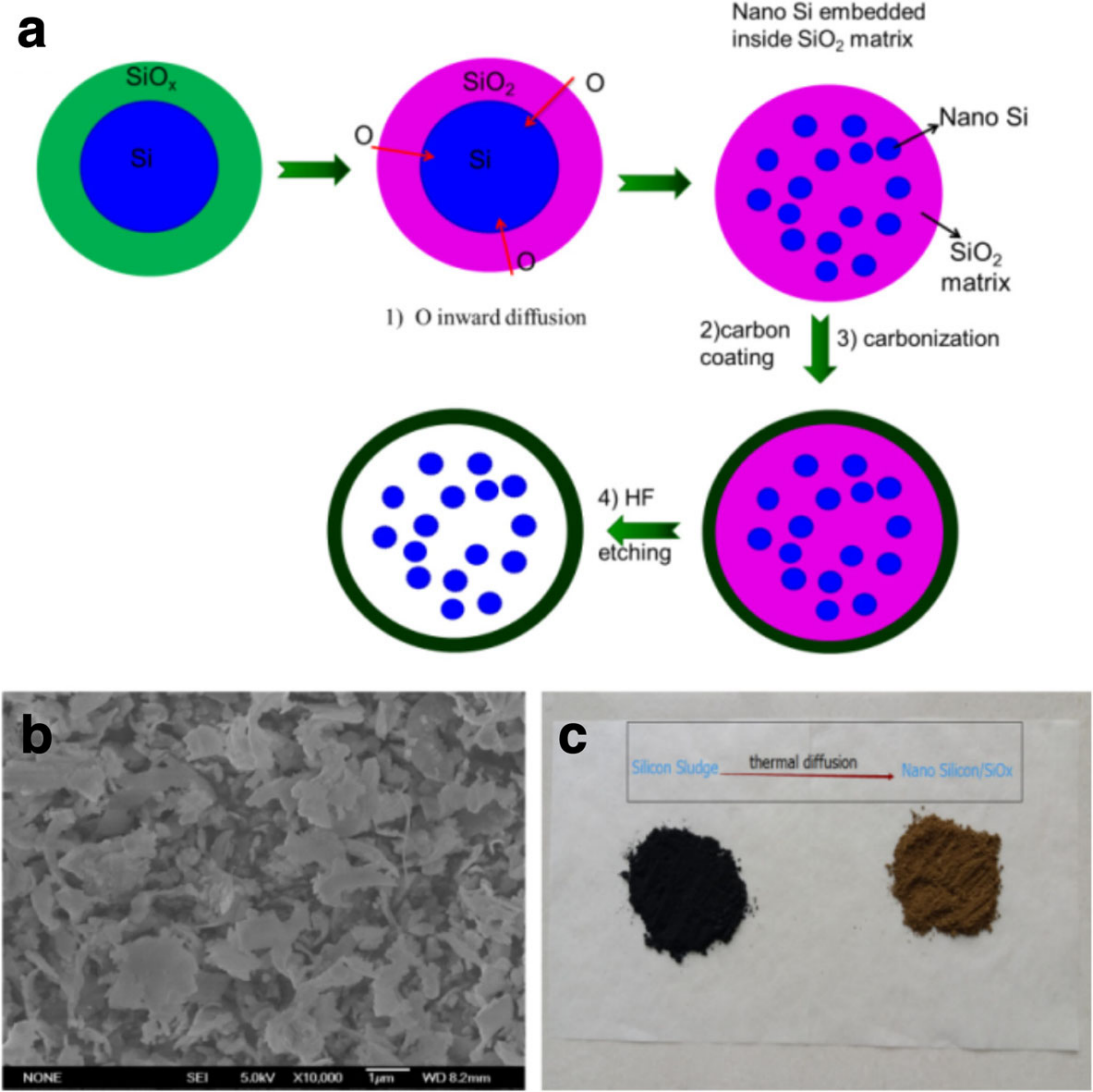
**a** Schematic illustration for nano-Si/SiO_x_ formation and further Si/C yolk/shell structure formation. **b** SEM image of nano-Si/SiO_x_ sample. **c** Real sample images

Firstly, Si sludge from a multi-wire slicing process, which was provided by LONGI Silicon Materials Corp., was cleaned with HCl and ethane to remove impurities. Because this crystalline Si wafering process is a mechanical cleavage process occurring along the tetrahedral Si, the Si sludge almost forms the flake shape. Meanwhile, most photovoltaic Si wafer has preferred p-type boron doping, this could help the conductivity as anode material for lithiation/delithiation [13]. Black Si sludge was annealed in alumina crucibles under an air atmosphere at 550 °C for 10 h to obtain a sufficient oxygen inter-diffusion process and convert to brownish nano-Si/SiO_x_ sample. Afterward, 1 g annealed sample was dispersed in 240 mL of deionized water and 0.8 mL of NH_3_•H_2_O (Aladdin, 28%). After vigorously stirring for 20 min, 400 mg of resorcinol and 0.56 mL of formaldehyde-water solution (37 wt.%) were added into the very diluted mixture and stirred overnight, to coat a resorcinol-formaldehyde (RF) resin layer on the surface of nano-Si/SiO_x_ sample. The RF layer was then converted to a carbon layer under Ar at 850 °C for 2 h with a heating rate 5 °C/min. Finally, the composites were dispersed in 10 wt% of HF solution to remove the SiO_x_ part, and Si/C yolk/shell structure can be obtained, detailed process is referred in Reference [14], a control sample was prepared in same procedure using Si sludge, without the process of oxygen inward diffusion to form nano-Si/SiO_x_ part. The whole process was shown in Fig. 1a, and this nano-Si/SiO_x_ sample has a flake shape as seen in Fig. 1b SEM image. The resulting flakes were brownish, as seen in Fig. 1c.

For electrochemical characterization, a 1 MLiPF6 in EC/DEC/DMC 1:1:1 (volume ratio) as the electrolyte, and a Celgard 2400 membrane was used as the separator. The working electrodes were prepared by mixing 80 wt% active materials (Si/C), 10 wt% acetylene black, and 10 wt% PVDF dissolved in an NMP solution. The cells were charged and discharged on a land test system (LAND CT2001A) in a voltage window of 0.01–2.5 V at a rate of 100 mA/g. Cyclic voltammetry (CV) and electrochemical impedance spectroscopy (EIS) were conducted on an electrochemical workstation (CHI660C) at a scanning rate of 0.5 mV/s. EIS measurements were recorded by applying an AC voltage of 10 mV over a frequency range of 10^5^ to 0.01 Hz.

## Results and Discussion

**Fig. 2:**
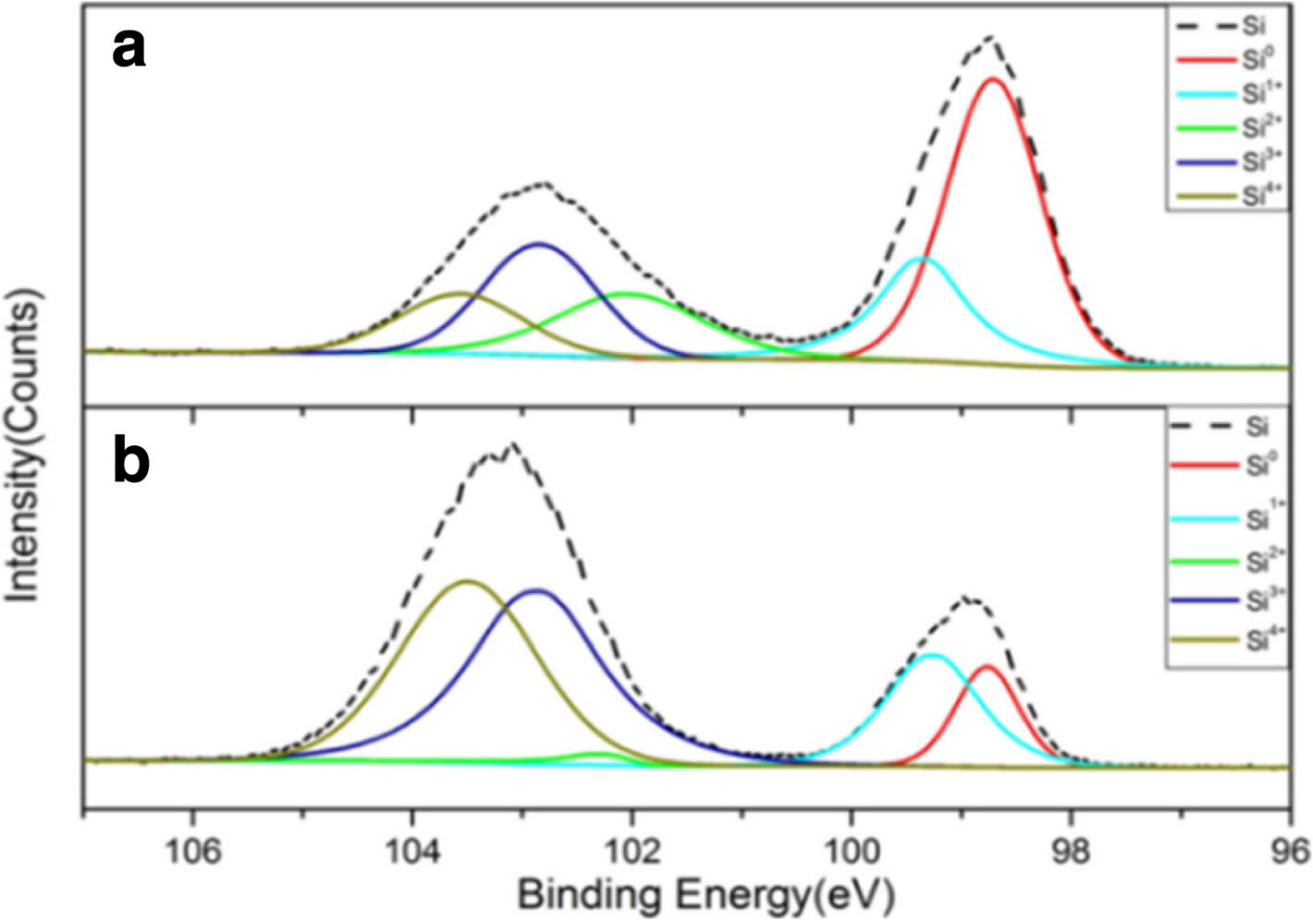
Si 2p XPS spectra (dash line) and their deconvolution fitting into five chemical states (from Si0 to Si4+, color lines) of the Si 2p for Si sludge in **a** and nano-Si/SiOx sample in **b**, respectively

X-ray photoluminescence spectroscopy (XPS) measurements were conducted, as shown in Fig. 2. The Si 2p spectra can be de-convoluted into five valence states: Si^0^, Si^1+^, Si^2+^, Si^3+^, and Si^4+^ [15]. Figure 2a shows the XPS results of the Si 2p spectra for the original Si sludge, these for the nano-Si/SiO_x_ samples after diffusion are shown in Fig. 2b, and these results confirm the obvious phase change from the Si sludge.

In particular, the pure Si^0^ is clearly reduced, and various sub-oxide states become more pronounced. The various Si sub-oxide states can actually be considered to be stoichiometrically different for the Si and SiO_2_ mixture. The amounts of the Si oxidation states for each sample are summarized in Table 1.

**Table 1:** Percentages of Si oxidation states by XPS measurements in Fig. 2. Obvious enhancements of various Si oxidation states can be founded from (a) to (b)

Sample	Si^0^ (%)	Si^1+^ (%)	Si^2+^ (%)	Si^3+^ (%)	Si^4+^ (%)
(a)	41.7	14.7	4	23.1	16.5
(b)	9.1	16.4	1.4	37.7	35.4

Inter-diffusion is a thermodynamically preferred process for Si wafer-based semiconductor industry [16]. Here, the O interstitial diffusion acted as a knife, cutting the Si core into nano-fragments, and diffused O formed SiO_x_ with the rest of the neighboring Si parts. The volume ratios of the nano-Si:SiO_x_ actually relied on the diffusion conditions such as the amount of O participated in the thermal diffusion [17]. Table 2 lists the weight percentages of Si and O in nano-Si/SiO_x_ particles examined by X-ray fluorescence (XRF), which confirms the oxygen content of produced SiO_x_ increases as long as the thermal oxidation time increase.

**Table 2:** Percentages of Si and O of different thermal oxidized Si sludge samples with different oxidation times. All data are before normalization

Oxidation time (h)	5	7.5	10
Si weight percent (%)	71.05	65.48	60.12
O weight percent (%)	28.42	33.96	39.28
Si:O (mole ratio)	1:0.7	1:0.91	1:1.14

**Fig. 3:**
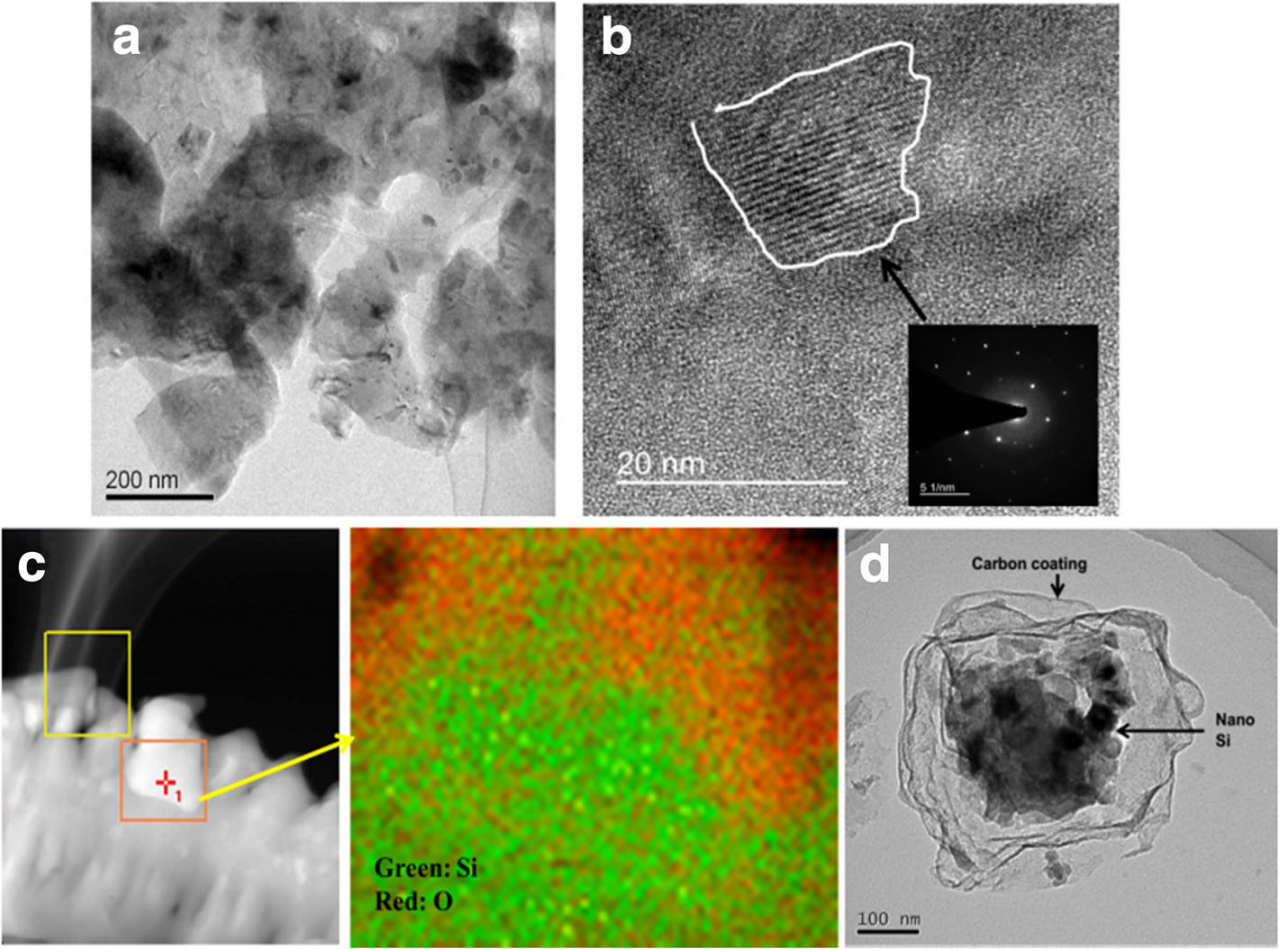
TEM characterizations for the nano-Si/SiO_x_ matrix sample. **a** Particle morphology. **b** Particle observation under high magnification shows the crystalline Si core and amorphous oxide surrounding. **c** STEM/EDX elemental mapping prove the miscible feature of Si and O. **d** Image of Si/C yolk/shell structure

Transmission electron microscopy (TEM) was conducted as shown in Fig. 3a, b. The lattice structure of the nano-Si/SiO_x_ sample was confirmed using selected area electron diffraction (SAED). The elemental mappings of the nano-Si/SiO_x_ sample and final Si/C yolk/shell sample were evaluated using high-resolution STEM/EDX (Tecnai G2 F20 S-TWIN), as shown in Fig. 3c, d, respectively. Figure 3a shows that nano-Si/SiO_x_ specimen retained the flake shape of the original Si sludge, and under high magnification, as shown in Fig. 3b, we found that polycrystalline Si phases were dispersed inside the amorphous matrix, existing as either nano-Si islands or nano-chains. The mechanism of this nano-Si/SiO_x_ structure can be explained by the oxygen inward diffusion along the interface [18]. The air atmosphere acted as an O reservoir; there was no energy barrier to O interstitial diffusion across the Si/Si oxide interface; the O could continuously penetrate the Si until reaching saturation. It can be observed in the element mapping of Fig. 3c that the O is miscible with Si, and a certain region with rich Si content indicates a nanocrystalline Si site. After removing oxide, clear yolk/shell Si/C composite structure can be seen in Fig. 3d. Moreover, HF removing SiO_2_ is not environmental friendly, but experience learned from semiconductor industry could provide a low cost and green method to recycle HF from fluorosilicic acid, such as ammoniated precipitated [19].

**Fig. 4:**
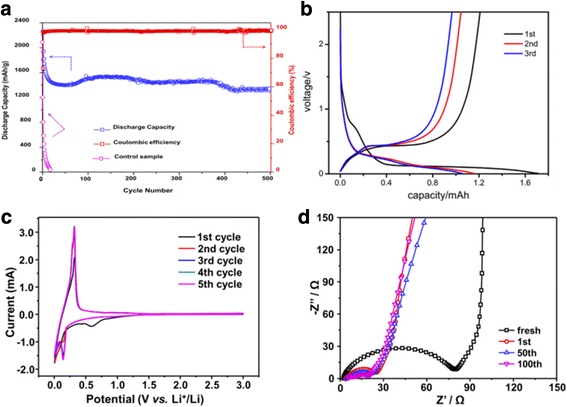
Electrochemical performance. **a** Discharge capacity and coulomb efficiency cycling performance of this Si/C composite at rate of 100 mA/g and comparison with control sample. **b** Voltage profile of this Si/C composite at 1st, 10th, and 100th cycles. **c** CV curves of the first 5 cycles of this Si/C composite electrode. **d** Nyquist plots of the Si/C composite electrodes after several tens cycles at the discharged state

Electrochemical tests were shown in Fig. 4, performed half-cells (this Si/C composite was used nano-Si/SiO_x_ with Si:O = 1:0.85, as raw material) showed an excellent performance with 500 cycles. Its capacity still remained above 1250 mAh/g, and the average Coulombic efficiency of the cells was up to 99.5% in Fig. 4a. In contrast, the controlled sample was totally fail charge/discharge process after less than 20 cycles. Figure 4b provides the voltage profiles of the Si/C electrode at the 1st, 10th, and 100th cycles at a rate of 100 mA/g between 0.01 and 2.5 V. An irreversible plateau is observed at around 0.75 V at first cycle, which can be attributed to the formation of a SEI film on the surface of the Si/C electrode. For all of the cycles, a plateau is shown at approximately 0.5 V, which was caused by the de-alloying of the Li-Si. The cycle voltammetry (CV) results shown in Fig. 4c are also the typical electrochemical features of Si [20]. The peak below 0.2 V in the negative scan and the peaks ~ 0.4 V in the positive scan, respectively, correspond to Li alloying and de-alloying process with Si. These peaks retain with cycles, implying that the Si parts are stable and accessible to Li ions. C coating provides a rapid lithium transport pathway, which may account for the very small cell impedance (Fig. 4d), as compared to most reported Si/C composite structure.

## Conclusions

In summary, the abundant Si sludge was utilized to produce a novel nano-Si/SiO_x_ using simple oxygen thermal diffusion as a precursor. After further coating a carbon layer and HF etching, a Si/C yolk/shell structure was obtained, which showed an excellent electrochemical performance for LIB anode. We found a simple, environmentally friendly way by converting the large of waste Si sludge into a valuable anode material for LIB applications. This “kill two birds with one stone” work will be beneficial for both the PV and LIB industries.
